# Lack of Direct Correlation between Biofilm Formation and Antimicrobial Resistance in Clinical *Staphylococcus epidermidis* Isolates from an Italian Hospital

**DOI:** 10.3390/microorganisms10061163

**Published:** 2022-06-06

**Authors:** Davide Carcione, Gabriella Leccese, Gianmarco Conte, Elio Rossi, Jari Intra, Alice Bonomi, Simona Sabella, Massimo Moreo, Paolo Landini, Matteo Brilli, Moira Paroni

**Affiliations:** 1 Laboratory of Microbiology and Virology, IRCCS San Raffaele Scientific Institute, 20132 Milan, Italy; carcione.davide@hsr.it; 2 Department of Laboratory Medicine, IRCCS Centro Cardiologico Monzino, 20138 Milan, Italy; simona.sabella@ccfm.it (S.S.); massimo.moreo@ccfm.it (M.M.); 3 Department of Bioscience, University of Milan, 20133 Milan, Italy; gabriella.leccese@unimi.it (G.L.); gianmarco.conte@unimi.it (G.C.); elio.rossi@unimi.it (E.R.); paolo.landini@unimi.it (P.L.); 4 Clinical Chemistry Laboratory, University of Milano-Bicocca, Azienda Socio Sanitaria Territoriale di Monza ASST-Monza, San Gerardo Hospital, Via Pergolesi 33, 20900 Monza, Italy; j.intra@asst-monza.it; 5 Unit of Biostatistics, IRCCS Centro Cardiologico Monzino, 20138 Milan, Italy; alice.bonomi@ccfm.it

**Keywords:** *Staphylococcus epidermidis*, biofilm, crystal violet, antibiotic resistance polysaccharide intercellular adhesin (PIA), Congo red agar

## Abstract

*Staphylococcus epidermidis* is an opportunistic pathogen and a frequent cause of nosocomial infections. In this work, we show that, among 51 *S. epidermidis* isolates from an Italian hospital, only a minority displayed biofilm formation, regardless of their isolation source (peripheral blood, catheter, or skin wounds); however, among the biofilm-producing isolates, those from catheters were the most efficient in biofilm formation. Interestingly, most isolates including strong biofilm producers displayed production levels of PIA (polysaccharide intercellular adhesin), the main *S. epidermidis* extracellular polysaccharide, similar to reference *S. epidermidis* strains classified as non-biofilm formers, and much lower than those classified as intermediate or high biofilm formers, possibly suggesting that high levels of PIA production do not confer a particular advantage for clinical isolates. Finally, while for the reference *S. epidermidis* strains the biofilm production clearly correlated with the decreased sensitivity to antibiotics, in particular, protein synthesis inhibitors, in our clinical isolates, such positive correlation was limited to tetracycline. In contrast, we observed an inverse correlation between biofilm formation and the minimal inhibitory concentrations for levofloxacin and teicoplanin. In addition, in growth conditions favoring PIA production, the biofilm-forming isolates showed increased sensitivity to daptomycin, clindamycin, and erythromycin, with increased tolerance to the trimethoprim/sulfamethoxazole association. The lack of direct correlation between the biofilm production and increased tolerance to antibiotics in *S. epidermidis* isolates from a clinical setting would suggest, at least for some antimicrobials, the possible existence of a trade-off between the production of biofilm determinants and antibiotic resistance.

## 1. Introduction

*Staphylococcus epidermidis* is a coagulase-negative, Gram-positive coccoid, catalase positive, and facultative anaerobe bacterium. Normally, it is a symbiont and is highly abundant on the human skin or mucosa [1]. However, *S. epidermidis* can easily switch from a symbiotic to an invasive lifestyle, causing virulence and colonizing the human body. Due to its ability to colonize medical and prosthetic devices, *S. epidermidis* is one of the most common causes of nosocomial blood infections. Indeed, subjects with contact lenses, prosthetic joints, cardiac devices, urinary and central venous catheters, endotracheal tubes, orthopedic devices as well as individuals treated with intravenous drugs are at higher risk of infection with *S. epidermidis* and other coagulase-negative staphylococci [2,3,4]. The infection usually occurs when the bacteria migrate from the human’s skin to the medical device, causing symptoms such as inflammation, erythema, or purulence [2,3,4]. The European Center for Disease Prevention and Control (ECDC) Annual Epidemiological Report for 2017 on surgical site infections showed that among the 4727 microorganisms identified, 51.6% were Gram-positive cocci, particularly 11.0% coagulase-negative staphylococci [5]. In coronary artery bypass graft, hip prosthesis surgery, knee prosthesis surgery, and laminectomy, coagulase-negative staphylococci represented the third cause of surgical infection [5]. Moreover, catheter-related staphylococcal infections are among the most common nosocomial infections, accounting for significant morbidity and mortality [6,7].

At present, antimicrobial treatments for *S. epidermidis* are complicated by the increasing frequency of resistant isolates: indeed, resistance to methicillin is reported in more than 80% of coagulase-negative staphylococci, while the type and severity of the infection, and the source of the infection can also hinder the success of antibiotic treatment [1,2,3]. Furthermore, the ability to form biofilms, often highly recalcitrant to antibiotic therapies, is considered as a major pathogenesis determinant in *S. epidermidis* [8]. Biofilms consist of communities of microbial cells attached to a solid surface that produce a matrix composed of secreted cellular polymeric substances that can create a shield against cellular insults for the bacterial population [2,9]. The first observations obtained from electron microscope analysis of explanted Spitz–Holter shunts and central venous catheters showed that *S. epidermidis* was organized into adherent, thick, and multilayered bacterial agglomerates embedded in an amorphous, bacterially-derived extracellular matrix [2,3,10]. Numerous studies have identified and further characterized the factors specifically involved in primary attachment and in mediating bacterial–surface interactions. In *Staphylococcus* spp., the polysaccharide intercellular adhesin (PIA), also called β-1-6-linked N-acetylglucosamine (PNAG), is produced by enzymes encoded by the *ica* operon and is the most widely known intercellular adhesin involved in biofilm formation and immune evasion [2,11,12,13,14]. Biofilm formation in *S. epidermidis* is a multistep process that involves several cell surface proteins with different functions in bacterial–extracellular matrix interactions such as the peptidoglycan-hydrolase AtlE involved in the binding to unmodified polystyrene, the accumulation-associated protein (Aap) and the serine-aspartate repeat proteins (Sdr) involved in the adhesion to surface, and extracellular DNA involved in the primary attachment to glass surfaces and in the stabilization of biofilms [2,6,11,12,13]. Biofilm formation is often controlled by quorum sensing mechanisms [15]: in Staphylococci, the agr quorum sensing system can promote the production of virulence factors while downregulating the adhesion factor production [16].

Since growth as biofilms protects *S. epidermidis* cells from attacks by host defenses and can hamper antibiotic treatment, thus resulting in longer and more severe infections [17], we investigated the possible correlations between proficiency in the biofilm formation and antibiotic susceptibility patterns in clinical *S. epidermidis* strains isolated from various biological materials of cardiac surgery patients at the Monzino IRCCS Cardiology Center. Fifty-one isolates derived from peripheral blood, catheters, and wounds were characterized for their ability to produce PIA and biofilm, and their antibiotic susceptibility was tested by the broth microdilution method. The relationships between the isolation source, biofilm- and slime-producer strains, and antibiotic susceptibility were studied through the use of multivariate regression models (GLM).

Our data show that that *S. epidermidis* isolated from catheters showed a higher ability to form biofilm than the isolates from wounds or other tissue. Intriguingly, slime-negative and biofilm non-producer *S. epidermidis* clinical strains not only failed to display increased sensitivity to antibiotics compared to the biofilm/slime positive strains, but were even more resistant to some antimicrobials (namely, gentamicin and tetracycline for slime-negative strains and, erytromycin, daptomycin, and clindamycin for the biofilm non-producer strains, respectively), possibly suggesting a sort of trade-off between the biofilm formation and antibiotic resistance in clinical isolates.

## 2. Materials and Methods

### 2.1. Bacterial Strains

A total of 51 *S. epidermidis* isolates from either peripheral blood (*n* = 31, Group A) or catheters (*n* = 8, Group B) or wounds (*n* = 12, Group C) from patients hospitalized at the Monzino IRCCS Cardiology Center were analyzed. In order to assess the influence of media composition on bacterial growth, biofilm formation, exopolysaccharide production, and antimicrobial susceptibility, all *S. epidermidis* strains were grown in four different media, namely, trypticase soy broth (TSB Difco^TM^, Franklin Lakes, NJ, USA), TSB with 1% glucose (TSB_GLU_), brain heart infusion (BHI, Difco^TM^), and BHI with 2% sucrose (BHI_SUC_).

*S. epidermidis* reference strains possessing either moderate (ATCC 35,983, strain designation RP12) or high biofilm-forming ability (ATCC 35,984, strain designation RP62A) as well as a non-biofilm producer (ATCC 155, strain designation AMC 263) were also included in our study as controls [18,19,20].

### 2.2. Organism Identification and Antimicrobial Susceptibility Testing

The identification of *S. epidermidis* isolates was performed with the VITEK^®^ 2 automated system (bioMérieux, Craponne, France) using Card VITEK^®^ 2 GP. The antimicrobial susceptibility testing of *S. epidermidis* strains, grown in the different media described above, was determined by the broth microdilution method Sensititre^TM^ Gram Positive EUSTAPF Plate (Thermo Fisher, Waltham, MA, USA). The antibiotics tested were: ceftaroline, clindamycin, daptomycin, erythromycin, fusidate, gentamicin, levofloxacin, linezolid, moxifloxacin, rifampin, teicoplanin, telavancin, tetracycline, tobramycin, trimethoprim/sulfamethoxazole, and vancomycin. The minimum inhibitory concentrations (MICs) were interpreted using the criteria recommended by the EUCAST, European Committee on Antimicrobial Susceptibility Testing (http://www.eucast.org/ accessed on 1 July 2020) [21].

### 2.3. Visualization and Quantification of In Vitro Biofilm Formation

Biofilm formation was quantified using the crystal violet staining of bacteria attached to the polystyrene surface of a 96-well microtiter plate [22]. Briefly, overnight bacterial culture, grown in various conditions (TSB, TSB_GLU_, BHI, and BHI_SUC_), were diluted to OD_600_ = 0.02 and incubated in respective media in triplicate (200 μL/well) in a 96-well round bottom plate for 16 h at 37 °C in the stationary condition. Next, the plates were washed with phosphate-buffered saline (PBS) to remove unattached strains and then stained with 1% CV solution for 20 min at room temperature. After washing, adherent stains were dissolved in a solution containing 96% ethanol in water and quantified at 550 nm in a microplate reader (SAFAS-MP96, Monaco). The adhesion index (AI) was calculated as OD_550_(CV)/OD_600_ (planktonic culture). The experiment was performed in triplicate and repeated three times, the data were then averaged and the standard error medium (SEM) was calculated. The isolates were classified as negative when the cut-off value corresponded to the non-adherent value of reference strain ATCC155 (AI < 0.2), as positive when the cut-off value corresponded to the strongly adherent value of the ATCC35984 strain (AI > 1) or moderate when the cut-off value corresponded to the weakly adherent value of the ATCC35983 strain (0.2 < AI < 1). This range of AI values were determined according to the on OD_550_ (CV) cut-off values previously established by Oliveira and Cunha [23].

Visualization of the biofilm formation was performed growing *S. epidermidis* strains onto glass coverslip in 24-well plates [24]. Briefly, overnight bacterial culture, grown in TSB_GLU_, were diluted to OD_600_ = 0.02 and incubated in the same media for 16 h at 37 °C in stationary condition. Next, the biofilms were fixed with methanol, stained with a solution of Giemsa for 15 min at room temperature, and visualized under a light microscope.

### 2.4. Phenotypic Analysis of Polysaccharide Intercellular Adhesin (PIA) Production

Phenotypic characterization of bacteria-producing slime in terms of PIA production was assessed by the original CRA test [25] with some modification. Briefly, overnight bacterial culture, grown in various conditions (TSB, TSB_GLU_, BHI, and BHI_SUC_), were diluted to OD_600_ = 1 and spotted (3 μL) on BHI agar plates supplemented with 5% sucrose (Sigma-Aldrich, Darmstadt, Germany) and 0.08% Congo red (Sigma-Aldrich, Darmstadt, Germany). The plates were incubated either at 35 °C or 37 °C under aerobic conditions for 24 and 48 h [20,26]. Strains that displayed red/bordeaux colonies were considered as slime-negative results while black colonies with a dry crystalline consistency were considered as positive results according to the previously described classification [27,28]. The experiment was performed in triplicate and repeated two times.

### 2.5. Presence of aap and agrB Genes among Clinical S. epidermidis Isolates

The presence of *aap* and *agrB* genes was investigated on a subset of clinical *S. epidermidis* isolates selected on the basis of their biofilm proficiency and slime production. One bacterial colony of each isolate was inoculated into 50 μL of nuclease-free water and lysed by heating at 98 °C for 10 min. After centrifugation at maximum speed for 5 min, 3 μL of the collected supernatant were used as a template for PCR amplification, as previously described [29]. The primer sequences used in this study are listed in Supplementary Table S1.

### 2.6. Statistical Analysis

All of the statistical analysis described in the main text was performed in R. The functions used were: Fisher test, *t*-test, and GLM from the stats package, with default options where needed if not unless specified in the main text. The figures were obtained with Prism software (version 7; GraphPad Software, Inc., La Jolla, CA, USA) and the heatmap with the package pheatmap.

## 3. Results

### 3.1. PIA Production Correlates with Antibiotic Resistance and Biofilm Production in S. epidermidis Reference Strains

**Figure 1:**
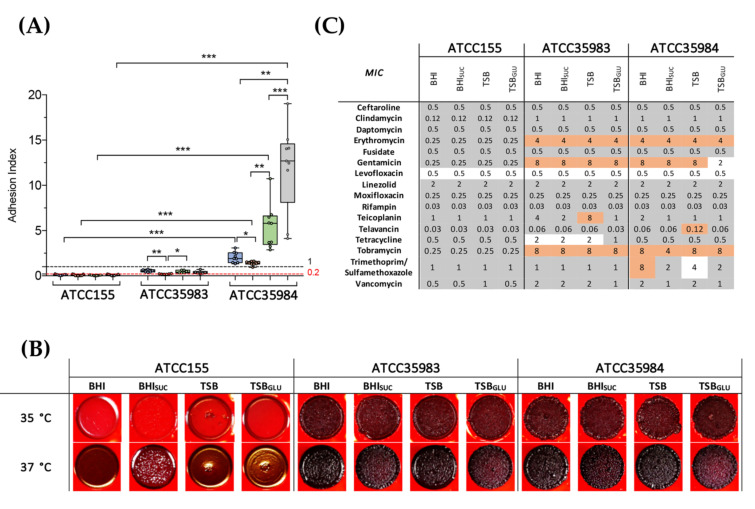
The phenotypic profile of *S. epidermidis* refence strains. (**A**) Biofilm formation determined with the CV assay of the indicated *S. epidermidis* reference strains in BHI in the presence or absence of 2% sucrose (BHI_SUC_), or TSB in the presence or absence of 1% glucose (BHI_SUC_). Data are represented with Box-plots showing each value as dots, the median as horizontal lines, and range with upper and lower quartiles of at least three independent experiments. Statistical significance for each condition was reported (* *p* < 0.05, ** *p* < 0.01, *** *p* < 0.001). (**B**) Qualitative detection of the slime formation on Congo Red agar (CRA) plates of the indicated refence strains at 35 °C (upper panel) and 37 °C (lower panel). (**C**) The MIC values of the *S. epidermidis* reference strains grown in different indicated media. Each experiment was performed in triplicate. Resistant strains or with a MIC value higher then MIC90 was reported in red; susceptible or with a MIC value lower then MIC90 was reported in grey; and susceptible-increased exposure in white.

The main goal of our work was to investigate the existence of significant relationships between biofilm formation, antimicrobial resistance, and the production of polysaccharide intercellular adhesin (PIA), supposedly a main determinant for biofilm formation, immune evasion, and overall virulence in *S. epidermidis* clinical isolates. To this aim, we first tested the behavior of the standard reference strains of *S. epidermidis* that differed for their ability to form biofilm, namely, ATCC155 (biofilm negative, isolated form skin) ATCC35983 (biofilm intermediate, isolated from blood), and ATCC35984 (biofilm proficient, isolated from a catheter), in our experimental conditions (Figure 1). As previous reports have showed a strong dependence of biofilm formation on the medium composition [30], we quantified surface adhesion by these reference strains in four different media, namely, trypticase soy broth (TSB), TSB with 1% glucose (TSB_GLU_), brain heart infusion (BHI), and BHI with 2% sucrose (BHI_SUC_). Supplementation of either glucose to TSB or sucrose to BHI (which already contains glucose) was aimed at assessing the role of either sugar as an environmental trigger in biofilm formation. Sucrose, in particular, is required to trigger PIA production in the solid medium [20]. As expected, the growth media appeared to strongly affect the biofilm formation, although in different ways (Figure 1A): interestingly, sucrose addition to BHI (i.e., in conditions promoting PIA production) strongly reduced the surface adhesion by both the moderate-biofilm producer ATCC35983 and the strong biofilm-producer ATCC35984 strains. In contrast, glucose supplementation to TSB showed strain-specific effects, reducing the adhesion in ATCC35983 while stimulating it in ATCC35984. We also observed that biofilm formation was much higher in the TSB than in the BHI for the latter strain.

Both the ATCC35983 and ATCC35984 strains displayed the black and dry wrinkled colony phenotype associated with PIA production on BHI_SUC_ supplemented with Congo red, independently of the media in which the overnight cultures used as inoculum were grown (Figure 1B). Only the non-biofilm former ATCC155 strain displayed a phenotype consistent with low PIA production, which appeared to be stimulated when ATCC155 pre-cultures were grown in BHI_SUC_ (i.e., in growth conditions favoring PIA production already in the culture used as for the inoculum). Low-level PIA production in ATCC155 was further reduced when the incubation temperature was lowered from 37 °C to 35 °C, unlike the other reference strains (Figure 1B). The lack of proficiency in biofilm formation by ATCC155 is consistent with poor PIA production; in contrast, the stronger biofilm phenotype displayed by ATCC35984, despite similar levels of PIA production as ATCC35983, would suggest that PIA might not the main determinant for surface adhesion in this strain.

Antibiotic susceptibility profiles suggested a correlation between PIA production and reduced sensitivity to the ribosomal inhibitors erythromycin, gentamicin, tobramycin, and clindamycin, and, to a lesser extent, to the glycopeptide antibiotic and cell wall inhibitor vancomycin (Figure 1C). For all of these antibiotics, the MIC profiles were largely independent of the growth media. Decreased antimicrobial sensitivity to tetracycline and teicoplanin, or to the association of trimethoprim/sulfamethoxazole was also observed in ATCC35983 and ATCC35984, respectively.

### 3.2. Dataset Clustering of Clinical S. epidermidis Strains According to Biofilm Formation

**Figure 2:**
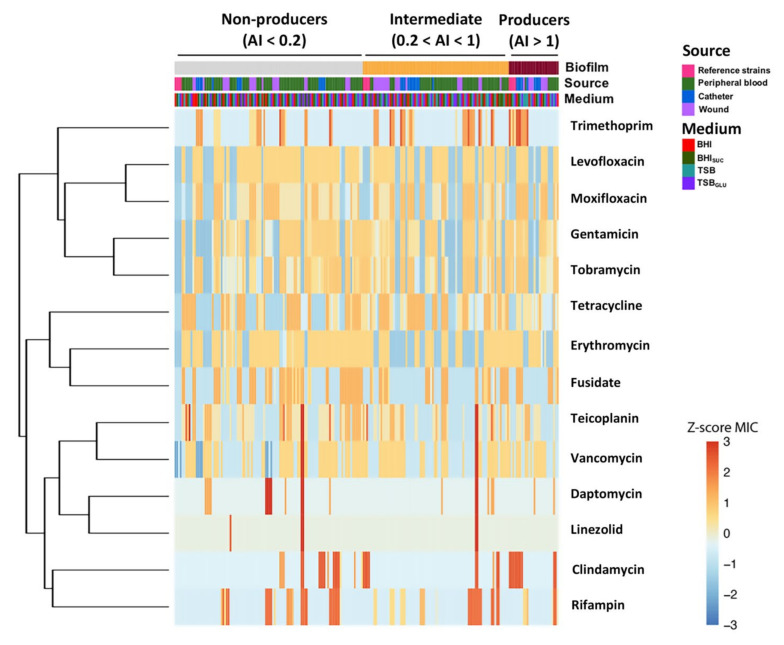
Heatmap of the antibiotic resistance profile of the *S. epidermidis* clinical isolates. *S. epidermidis* strains, isolated from the peripheral blood, catheters, or wounds of different patients and grown in different culture media, were grouped by their biofilm formation proficiency.

Once we defined the behavior of the standard reference strains in our experimental conditions, we set out to assess the existence of a significant relationship between the biofilm formation and antibiotic resistance profile among clinical *S. epidermidis* strains, collected from different anatomic sites (namely, peripheral blood, wound, and catheter) and grown in different media. To this aim, we performed hierarchical clustering based on the similarities of standardized MIC values: the results are represented as a heatmap, where the strains (columns) are sorted on the basis of their biofilm proficiency (adhesion index, first row) and clustered according to the reciprocal similarities in the MIC standardized profiles (Figure 2).

Our data demonstrated the absence of differentiated clusters according to the growth media, indeed, samples derived from the same *S. epidermidis* strain were mostly grouped together, suggesting that the MIC profile did not change significantly in different culture media (third row). Similarly, there was not a strict association between the biofilm formation and specific MIC profiles (columns). Indeed, clinical isolates with high MIC values (Z-score > 1) were randomly distributed among the three clusters of biofilm formation proficiency. Finally, when we considered the clustering of the antibiotics (rows,) we observed that those belonging to the same class tended to be grouped together such as the quinolones levofloxacin and moxifloxacin, the aminoglycosides gentamicin and tobramycin, and the glycopeptides teicoplanin and vancomycin, in agreement with the existence of cross-resistance mechanisms.

### 3.3. Correlation between PIA, Biofilm Production and Isolation Source among Clinical S. epidermidis Strains and Characterization of Adhesion Determinants

**Figure 3:**
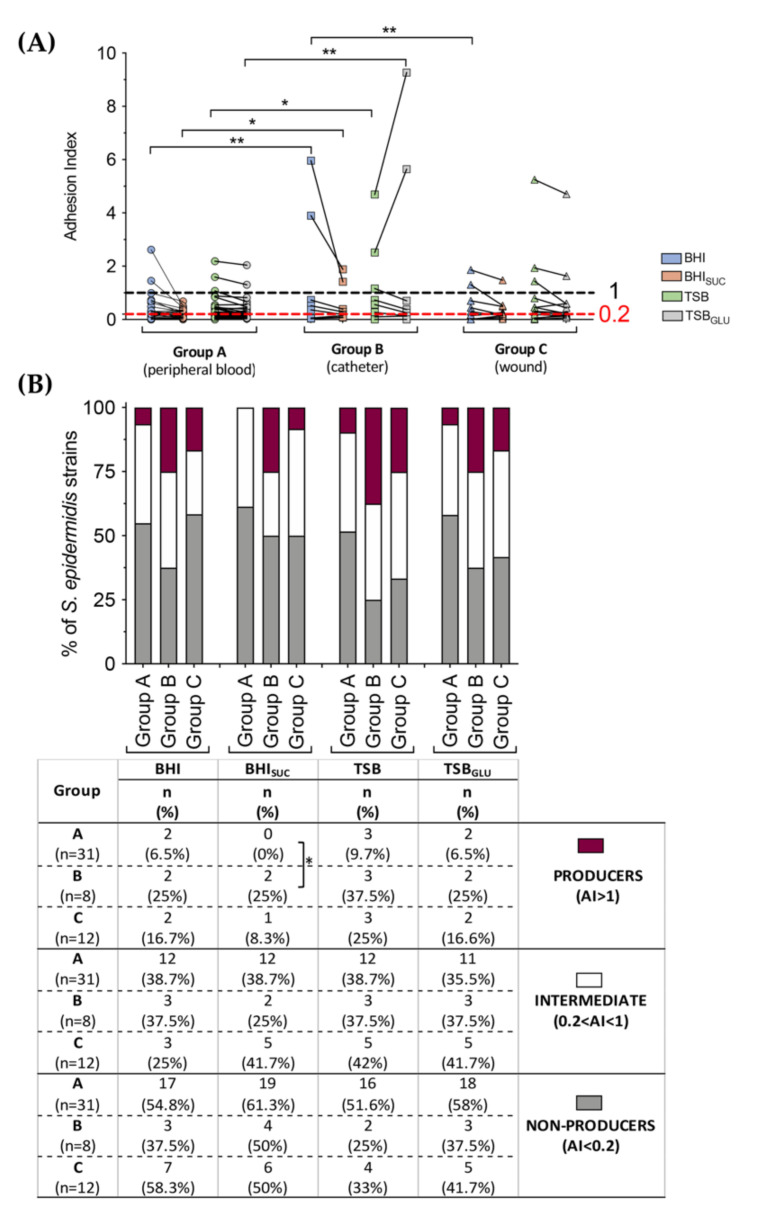
The biofilm formation of clinical *S. epidermidis* isolates. (**A**) Adhesion index, determined with the CV assay, of the clinical *S. epidermidis* strains isolated from the peripheral blood (Group A), catheters (Group B), or wounds (Group C) in different growth media as indicated. Data are represented with dot-plots showing the median value of at least three independent experiments of each isolate grown in BHI ± SUC and TSB ± GLU. Statistical significance analyzed with one-way ANOVA was reported (* *p* < 0.05, ** *p* < 0.01). (**B**) Percentage and number of strains classified according to the adhesion index (AI) value in strong biofilm-producers (AI > 1; purple), moderate biofilm-producers (0.2 < AI < 1; white), and non-producers (AI > 1; light gray) among the *S. epidermidis* clinical isolates derived from peripheral blood (Group A), catheters (Group B), or wounds (Group C). Statistical analysis performed with Fisher’s exact test for each condition was reported (* *p* < 0.05).

In order to compare in more detail the biofilm properties of the *S. epidermidis* clinical strains isolated at the IRCCS Centro Cardiologico Monzino, we stratified their adhesion index according to their isolation site (namely, peripheral blood, wound, and catheter) and to the growth medium. Our analysis revealed a significantly higher proficiency in the biofilm formation by isolates from catheters (Group B) compared to the peripheral blood (Group A), regardless of the growth media (Figure 3A). Indeed, the strains isolated from the catheters reached an average adhesion index (AI) of 0.439 in BHI and of 0.644 in TSB when compared to strains belonging to Group A (0.189 and 0.185 in BHI and TSB, respectively) and Group C (wound; 0.168 and 0.373 in BHI and TSB, respectively). Moreover, similar to what was observed with the ATCC reference strains (Figure 1A), the presence of sucrose in the growth medium negatively influenced the biofilm formation of clinical strains, while the addition of glucose to the TSB medium further enhanced the adhesion index exclusively in isolates from catheters already highly proficient in biofilm formation. The stratification of clinical strains performed according to the cut-off value described in Section 2 revealed a smaller, but not statistically significant (Fisher’s exact test) percentage of biofilm-producer isolates (AI > 1) from the peripheral blood than from the catheters and wounds (Figure 3B); the few biofilm-proficient isolates from the catheters showed a clearly significant higher capacity to adhere and form biofilm compared to the biofilm-positive isolates from the peripheral blood or wounds (Figure 3A).

**Figure 4:**
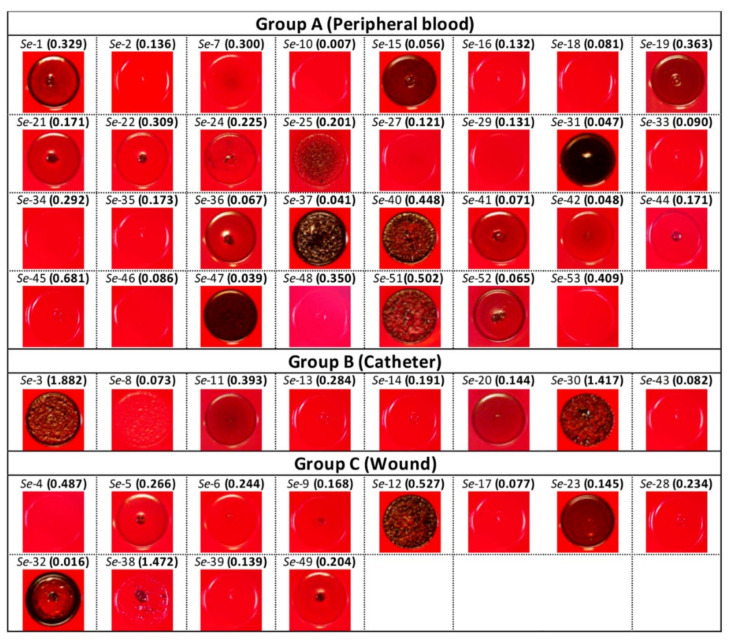
Relationships between the PIA expression and biofilm formation among the *S. epidermidis* clinical isolates. Qualitative detection of the slime formation on the Congo Red agar (CRA) plates of the *S. epidermidis* clinical strains grown at 37 °C subdivided according to their isolation source. The name of each clinical strain and, in brackets, its AI value obtained in BHI_SUC_, are reported above the colonies.

Phenotypical analysis on the CRA medium at 35 °C showed that only a small minority of the isolates were proficient in PIA production: indeed, only four slime-positive strains, with rough or opaque reddish-black colonies characteristic of PIA production, were found among the isolates from the peripheral blood (namely *Se*-31, *Se*-37, *Se*-40, and *Se*-51), two among the isolates from the catheters (*Se*-3 and *Se*-30), and two among the isolates from the wounds (*Se*-12 and *Se*-32) (Figure 4). Thus, PIA production did not strictly correlate with biofilm production, which, for some isolates, was even inhibited by growth in the BHI_SUC_ medium (Figure 3). Indeed, only for the isolates from the catheters did we observe a correspondence between a CRA phenotype indicative of PIA production and high levels of biofilm formation. Among the Group A isolates, only three of the 12 intermediate biofilm formers were PIA-producers, and one was among the non-biofilm producers (Figure 4). Only one isolate from the wounds showed a CRA-positive phenotype and was classified as an intermediate biofilm former. Thus, as already observed for the reference strains, and in line with previous observations in the literature [28,31], it appears that the clinical isolates here studied can rely on multiple biofilm determinants in addition to PIA.

**Figure 5:**
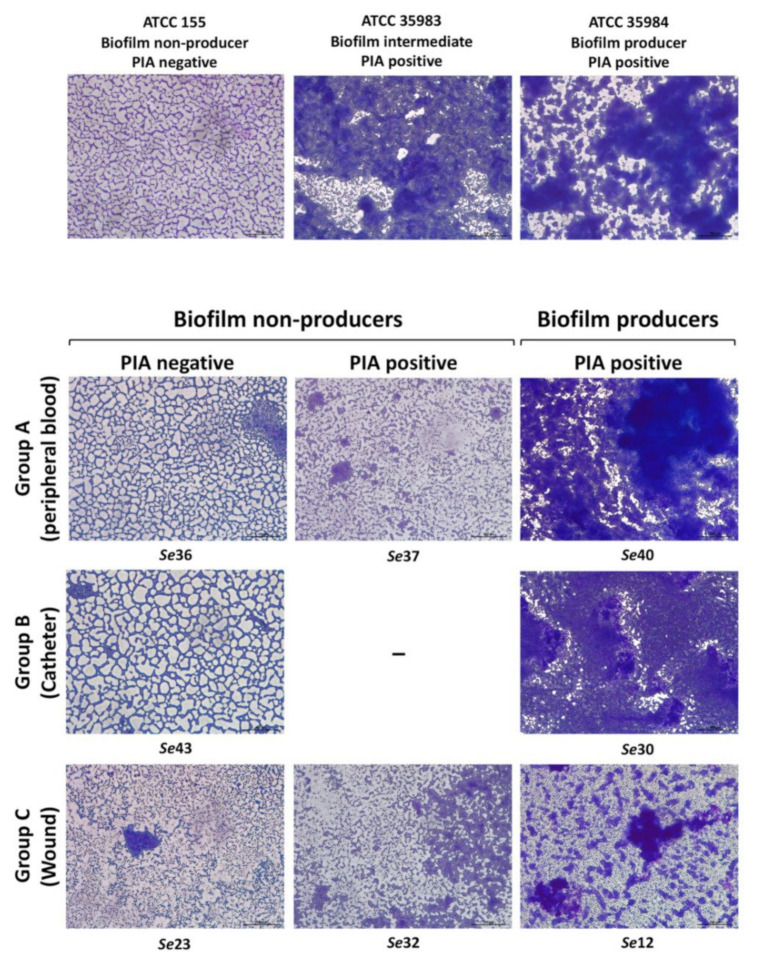
The visualization of the biofilm structure and matrix production among the *S. epidermidis* clinical isolates. Giemsa staining of the biofilms of the *S. epidermidis* reference strains (upper panel) and clinical isolates (lower panel) showing the different biofilm architectures formed by bacteria cultured on glass coverslips. The name of each clinical strain, subdivided according to their biofilm and PIA production, has been reported. Images were taken with a 200× magnification, the scale bars (bottom right corner of each image) represent 100 μm.

Although the crystal violet assay is a direct and reliable method for the assessment of biofilm formation, it does not provide any information on the biofilm structure and matrix production. Thus, in order to confirm our results, and to gather more information on the biofilm structure of the *S. epidermidis* clinical isolates, we carried out microscope observations following Giemsa staining on a selection of isolates from different sources, namely, the three biofilm non-producers/PIA negative (*Se*-36, *Se*-23, *Se*-43 from Groups A, B, C, respectively), two biofilm non-producers/PIA positive (*Se*-37, *Se*-32 from Groups A and C), and three biofilm producers/PIA positive isolates (*Se*-40, *Se*-30, *Se*-12 from Groups A, B, C, respectively) as well as the *S. epidermidis* reference strains tested in Figure 1. Microscopy observations performed on overnight cultures grown in TSB_GLU_ (Figure 5) clearly indicated how the biofilm-proficient isolates formed cell clusters, producing a significant amount of extracellular matrix readily stained by Giemsa. PIA-producers not proficient in biofilm formation produced a more limited and disorganized matrix, which seems to be totally absent in the biofilm non-producers/PIA negative isolates. Cell aggregation properties, assessed by cell sedimentation in the same overnight cultures used for microscopy observations (Supplementary Figure S1), seemed to reflect the production of the extracellular matrix by the different isolates as shown in Figure 5, and was also observed in PIA-producing isolates unable to form a biofilm on a solid surface.

**Figure 6:**
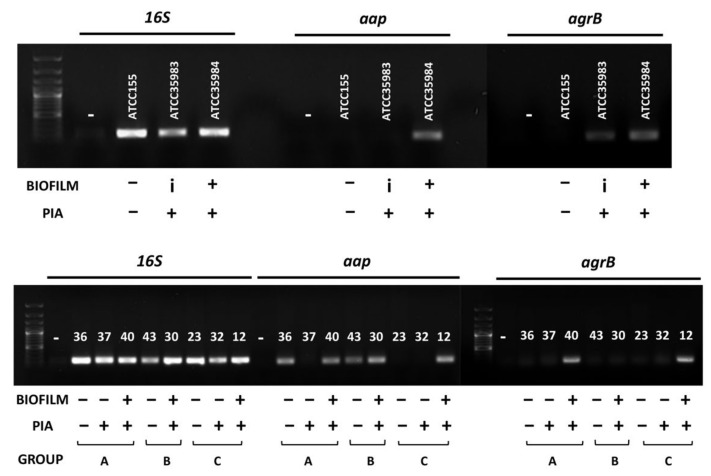
The PCR detection of *aap* and *agrB* genes in *S. epidermidis* clinical isolates. Agarose gel electrophoresis of PCR amplification of the 16S rRNA, *aap* and *agrB* genes in the *S. epidermidis* reference strains (upper panel) and clinical isolates (lower panel). The 100-bp DNA size ladder used as the molecular weight marker is shown on the left. The designation of each *S. epidermidis* strain/clinical isolate, and their phenotype with regard to biofilm formation, PIA production, and isolation source are indicated.

Our results are consistent with the notion that PIA, while able to promote cell aggregation to some degree, is not sufficient for proficient cell attachment to solid surfaces, which must thus rely on other adhesion factors. Several genetic factors can affect biofilm formation in *S. epidermidis* including the *aap* gene, encoding the accumulation-associated protein, a positive determinant for cell adhesion and biofilm formation, and the *agr* locus, a quorum sensing system known to positively affect *S. epidermidis* virulence while downregulating the expression of adhesion factor-encoding genes [2,32,33,34,35]. Neither the *agr* locus nor the *aap* gene are universally conserved in *S. epidermidis*, with several strains showing polymorphisms, or even deletion, of these loci [36,37]. Therefore, we verified the presence of these genes in the eight clinical isolates analyzed for their biofilm structure in Figure 5 by performing a PCR targeting the coding regions of the *agrB* and the *aap* genes. As shown in Figure 6, only the biofilm proficient isolates *Se*-40, *Se*-30, and *Se*-12 were both PIA producers and possessed the *aap* gene, which were, in contrast, missing in the PIA producers, the *Se*-37 and *Se*-32 isolates. Our results seem to suggest that the surface adhesion and formation of a fully developed biofilm require both the presence of functional *aap* alleles and PIA production as non-PIA producing isolates *Se*-36, *Se*-23, and *Se*-43 failed to form a biofilm regardless of the presence of *aap*. Surprisingly, the presence of the *agrB* gene, a negative determinant for adhesion factor production, was only detected in two out of the eight isolates tested (*Se*-40 and *Se*-12), both strong biofilm formers (Figure 6). These results were consistent with the reference strains, as the *aap* and *agrB* genes were only detected in the biofilm-proficient strain ATCC35984 (Figure 6).

### 3.4. Correlation between Biofilm Production, Isolation Source and Antibiotic Susceptibility in S. epidermidis Clinical Isolates

Biofilm formation is usually correlated with reduced antibiotic sensitivity, either by the linkage between biofilm determinants and antibiotic resistance genes or by the tolerance to antibiotics, resulting from the physiological changes in the bacterial cells due to the presence of the biofilm matrix. The antibiotic sensitivity patterns of the clinical isolates studied in this work are summarized in Supplementary Figure S2 (isolates grouped based on isolation site) and Supplementary Figure S3 (isolates grouped by biofilm proficiency). Overall, multidrug resistance was very common, as expected, particularly for the aminoglycoside, quinolone, and macrolide antibiotics.

In order to further evaluate the correlation performed with the hierarchical clustering (Figure 2) between biofilm production and antibiotic susceptibility in *S. epidermidis* clinical isolates, we resorted to a generalized linear model (GLM). GLM allows one to highlight the associations between the dependent and independent variables, while controlling at the same time for the confounding effects of additional variables. Therefore, GLM results in the calculation of coefficients for every predictor, but unlike classical regression, both categorical and numerical predictors can be combined. The corresponding variable can thus be positively or negatively associated with the dependent variable, in our case, the biofilm formation ability, via a positive or negative coefficient of significance. Additional variables can be introduced, and models of increasing complexity can be compared to understand whether the added regressors provide significant improvements to the performance of the model and therefore to understand if the introduction of more variables provides a significantly better model. In order to test a possible inverse correlation between antibiotic sensitivity and biofilm formation, the following models were constructed: biofilm adhesion index—medium + isolation source (M1), which was then compared with a model of biofilm—medium + isolation source + MIC values (M2 with MIC numerical values and M3 with binarized MICs into sensitive-resistant patterns only). Detailed information on the three models and their comparisons are reported in Supplementary Table S2; here, we performed the comparative analysis only with the best fitting model (M2) on the basis of the Akaike information criterion (Table 1). In this context, M2 highlights some significant associations between the biofilm formation and sensitivity to some antibiotics. In particular, our results showed a negative correlation between the biofilm formation and MIC values for levofloxacin and teicoplanin, and a positive correlation for tetracycline when all of the variables were considered at once (Table 1).

### 3.5. Antibiotic Resistance of S. epidermidis Clinical Isolates Does Not Positively Correlate with Ability to Form Biofilm or with PIA Production

In order to further analyze the correlations obtained with the GLM model (Table 1), we evaluated the impact of biofilm formation and PIA production on the antibiotic resistance patterns of the *S. epidermidis* clinical isolates. Since the GLM models demonstrated a complete independence among the different growth media and isolation source when all of the variables were considered simultaneously (Table 1 and Table S2), some significant differences in the BHI were observed among the biofilm-producer or non-producer strains (Supplementary Figure S3) when the variable of isolation source was removed. Here, we study the direct correlation between antibiotic susceptibility and either biofilm formation or PIA expression in two different growth media. More precisely, we correlated the antibiotic susceptibility of clinical *S. epidermidis* strains and biofilm formation in BHI, in which the clinical isolates displayed a somehow better ability to form biofilm (Figure 3A), while the antibiotic susceptibility and PIA production were compared in the medium used for the CRA assay (BHI_SUC_).

**Figure 7:**
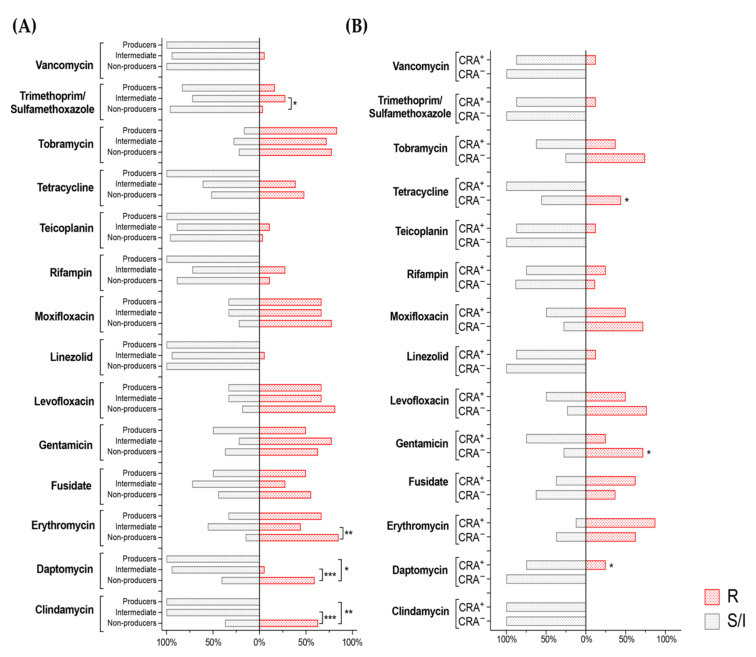
The relationships between antibiotic susceptibility, biofilm formation, and PIA expression among the *S. epidermidis* clinical isolates. (**A**) Prevalence of antibiotic resistance among the biofilm-producer, intermediate-producer, or non-producer clinical *S. epidermidis* strains. (**B**) Prevalence of antibiotic resistance among the CRA-positive (CRA^+^) and CRA-negative (CRA^−^) clinical *S. epidermidis* strains. Statistical significance analyzed with Fisher’s exact test for each condition was reported (* *p* < 0.05, ** *p* < 0.01, *** *p* < 0.001). R = Resistant (in red), S/I = Susceptible or increased exposure (in grey).

Our results indicate that none of the strong biofilm-producing isolates showed any degree of resistance to the antibiotics belonging to the glycopeptide class (vancomycin, teicoplanin, and daptomycin), or to rifampin. A more complex pattern emerged for antibiotics that interfere with protein synthesis: indeed, strong biofilm-producing isolates were sensitive to clindamycin, linezolid, or tetracycline, but often developed resistance to tobramycin (5 out of 6), gentamicin (3 out of 6), fusidate (3 out of 6), and erythromycin (4 out of 6) (Figure 7A). In contrast, low biofilm-forming strains were found to be sensitive only to vancomycin (27 out of 27), linezolid (27 out of 27), trimethoprim (26 out of 27), teicoplanin (26 out of 27), and rifampin (24 out of 27), while they showed reduced sensitivity, albeit to a different extent, to most antibiotics, in particular to daptomycin, clindamycin, and erythromycin in comparison to either the strong or moderate biofilm-producing strains (Figure 7A). The antimicrobial activity of ceftaroline and telavancin was assessed by MIC90 values since EUCAST has not yet determined the breakpoints. Evaluating all of the strains enrolled in this study, the MIC90 of ceftaroline and telavancin were 0.5 mg/L and 0.06 mg/L, respectively. Among the biofilm producers (nine strains with AI > 1), MIC90 of ceftaroline was 1 mg/L and 0.06 mg/L for telavancin.

Regarding the correlation between antibiotic susceptibility and slime-producing bacteria (CRA phenotype), since PIA expression was evaluated in BHI-agar supplemented with sucrose, we evaluated the impact of slime production on the antibiotic resistance patterns of the *S. epidermidis* clinical strains in the BHI_SUC_ medium (Figure 7B). Our results indicated that, in general, CRA negative strains exhibited a higher prevalence for multiple antibiotic resistance, in stark contrast to some studies reporting that slime production renders *S. epidermidis* strains highly resistant to antibiotics. Indeed, for the eight CRA-positive isolates, a higher prevalence was proven only for the resistance to fusidate (62.5% vs. 37.2% of CRA-negative strains), erythromycin (87.5% vs. 62.8%), and rifampin (25% vs. 11.6%) and statistically significant for daptomycin (25% vs. 0%, *p* = 0.022), while for all the other antibiotics, the slime-producing strain presented a lower prevalence of resistance (Figure 7B).

## 4. Discussion

Most *S. epidermidis*-related infections are acquired in hospital settings and are associated with the use of medical devices such as catheters. The ability of *S. epidermidis* to form a biofilm and their multi-drug resistance are widespread among clinical isolates and are thought to play a crucial role in their persistence in hospital settings. In particular, clinical isolates possess the *ica* operon, responsible for the production of PIA, the main extracellular polysaccharide in *S. epidermidis*, with a much higher frequency compared to the commensal strains [38]. However, the existence of a direct relationship between antibiotic resistance and the extent of slime/biofilm formation in *S. epidermidis* clinical strains remains a controversial issue. Indeed, some works have reported a higher incidence of antibiotic resistance among exopolysaccharide-forming *S. epidermidis* clinical strains, while other studies have displayed no difference in resistance rates between the biofilm-producer and non-producer *S. epidermidis* isolates [8,39,40,41,42,43].

In this work, 51 isolates from the Italian hospital Centro Cardiologico Monzino were characterized for their ability to form biofilms and to produce PIA, and for their antimicrobial sensitivity patterns. Since biofilm formation and adhesion factor production can be highly dependent on environmental conditions, we assessed these properties in four different growth media, and divided the isolates into three groups based on their isolation site (wound, peripheral blood, and catheter). The isolates were compared to three reference *S. epidermidis* strains different in their biofilm formation proficiency and PIA production (Figure 1). Interestingly, most of the 51 isolates, regardless of their isolation site, were either poor or intermediate biofilm formers (Figure 3) and poor PIA producers (Figure 4), as determined by the crystal violet and Congo red binding assays, respectively. The biofilm formation patterns did not show any major effect by the different growth media (Figure 3). However, the addition of sugars to the growth media seemed to affect the few biofilm proficient isolates in the opposite ways: indeed, sucrose resulted in a significant reduction in biofilm formation, while glucose further stimulated the biofilm production, at least in two biofilm-forming isolates from catheters (Figure 3A). This result would be consistent with previous reports that glucose can promote production of adhesion factors via the regulatory proteins CcpA [44,45]. In contrast, the addition of sucrose, which is added to a solid medium in order to increase the PIA production, negatively affects the biofilm formation in strong producers (Figure 3). This observation, together with the lack of a clear correlation between the levels of PIA production and biofilm formation (Figure 4 and Figure 5) would suggest that PIA is not a main factor in surface adhesion in the *S. epidermidis* clinical isolates. Indeed, a variety of adhesion factors such as proteinaceous adhesins [2,32,34] and even extracellular DNA [46,47] have been shown to be required for biofilm formation, and the *agr* quorum sensing system and other regulatory mechanisms presiding over biofilm formation have been characterized [13,48]. The PCR-mediated detection of adhesion genes on a subset of *S. epidermidis* clinical isolates (Figure 6) suggests that the *agr* locus, a negative regulator of adhesion factors, does not play an important role in biofilm regulation, as the *agrB* gene was only detected in strong biofilm formers, while it was missing in isolates incapable of forming a biofilm. In contrast, the presence of the accumulation-associated protein-encoding *aap* gene, together with PIA production, appears to be a determinant for biofilm formation, and allows for the formation of a mature biofilm structure (Figure 6). This would be consistent with the different roles played by polysaccharidic versus proteinaceous adhesins in cell aggregation and biofilm formation, already described for other microorganisms [49].

Multi-drug resistance constitutes a major problem in *S. epidermidis* nosocomial infections, often exacerbated in biofilm-forming strains [50,51]. The mechanisms linking the biofilm formation to reduced antibiotic sensitivity are complex and not fully understood, and they might include reduced permeability, altered metabolism, and cell physiology (e.g., persister cells), or direct association with the antibiotic resistance genes. Indeed, several reports have suggested that the transfer of conjugative plasmids and other DNA mobile elements is facilitated in bacterial biofilm settings [52,53,54,55]. Interestingly, while in the reference *S. epidermidis* strains the ability to form biofilm, and even PIA production, appeared to correlate with antibiotic resistance (Figure 1), such a pattern did not apply to the clinical isolates studied in this work. Indeed, the analysis of antimicrobial sensitivity patterns for the clinical isolates stratified as non-biofilm producers, intermediate, and strong biofilm formers, when grown in conditions favoring PIA production (Figure 7), showed a larger occurrence of antibiotic-resistant strains among the non- and intermediate biofilm formers for erythromycin, daptomycin, and clindamycin. Only for the association of trimethoprim/sulfamethoxazole could we detect a positive correlation between MIC and the ability to form biofilm. Likewise, no positive correlation emerged between PIA production and antibiotic resistance, with PIA producing isolates showing increased MIC for daptomycin alone, and lower MIC for tetracycline and gentamicin (Figure 7). When the correlation studies were extended to all of the growth conditions, as summarized in Figure 2, and analyzed with our GLM model (Table 1), an inverse correlation between the biofilm formation and antibiotic resistance emerged for levofloxacin and teicoplanin, while biofilm-forming isolates appeared to be less sensitive to tetracycline.

Taken together, our results suggest that a strong biofilm forming phenotype is not an essential feature in *S. epidermidis* isolates from a clinical setting, and that PIA production, although widespread, is not crucial for surface adhesion, which likely relies more on proteinaceous determinants such as Aap (Figure 5 and Figure 6). Finally, our observations indicate that biofilm formation does not strongly correlate with antibiotic resistance or tolerance, at least in standard MIC assays. In contrast, at least in some instances (Table 1, Figure 7), a biofilm forming phenotype seemed to correlate with increased sensitivity to some antibiotics. Increased sensitivity to some antibiotics in the biofilm proficient isolates might be due either to changes in the bacterial physiology, somehow linked to a higher production of adhesion factors such as differences in permeability or cell surface structures, or to evolutionary mechanisms, resulting in a trade-off between biofilm lifestyle and antibiotic resistance.

## Figures and Tables

**Table 1:** Antibiotics with a coefficient significantly different from 0 in model M2.

	Estimate	SE	*t*-Value	Pr (>|t|)
(Intercept)	−0.641	0.125	−5.136	6.75 × 10^−7^
Levofloxacin	−0.503	0.104	−4.835	2.69 × 10^−6^
Teicoplanin	−0.206	0.057	−3.605	3.95 × 10^−4^
Tetracycline	0.244	0.046	5.273	3.53 × 10^−7^

## Supplementary Materials

The following are available online at https://www.mdpi.com/article/10.3390/microorganisms10061163/s1. Table S1: Oligonucleotide primer sequences used in PCR gene amplification. Figure S1: Cell aggregation properties of the *S. epidermidis* clinical isolates. Figure S2: Relationships between the antibiotic susceptibility and isolation source among the *S. epidermidis* clinical strains. Figure S3: Relationships between the antibiotic susceptibility and biofilm formation among the *S. epidermidis* clinical strains. Table S2: Generalized linear model (GLM) comparison.
