# Supplementary material for: Lack of Direct Correlation between Biofilm Formation and Antimicrobial Resistance in Clinical Staphylococcus epidermidis Isolates from an Italian Hospital
Source: Microorganisms. 2022 Jun 6;10(6):1163. doi: 10.3390/microorganisms10061163 (PMC9230108; doi:10.3390/microorganisms10061163)
Supplement: Supplementary file 1 [file microorganisms-10-01163-s001.zip › microorganisms-1735467-supplementary.pdf]

Supplementary Table S1: Oligonucleotide primer sequences used in PCR gene amplification.

Target gene		Oligonucleotide primers sequence (5′ to 3′)	TM (ºC)	Amplicon size (bp)
16S	FW	GGGCTACACACGTGCTACAA	59.4	176
	RV	GTACAAGACCCGGGAACGTA	59.4	
aap	FW	ACCTACAACTTCAGAACCTGTGAAT	59.7	125
	RV	TAACCGTAGTTGGCGGTATATCT	58.9	
agrB	FW	AATTCGTTTAGGGATGCAGGT	55.9	142
	RV	ACCGTGTGCATGTCTCCTAAT	57.9	

Supplementary Figure S1

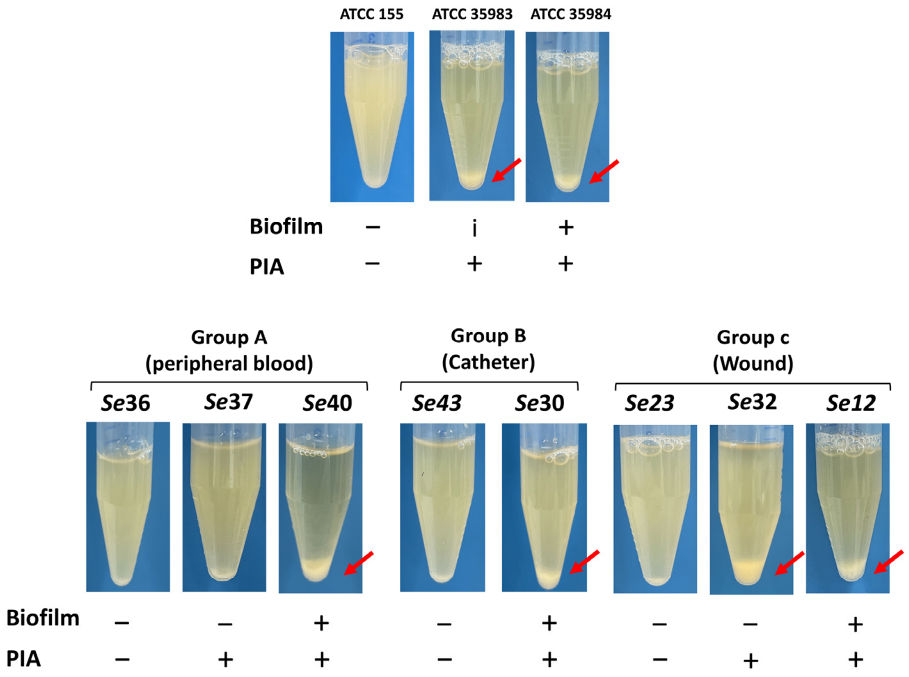

Figure S1: Cell aggregation properties of *S. epidermidis* clinical isolates.
Visual observation of cell aggregation of reference *S. epidermidis* strains (upper panel) and *S. epidermidis* clinical isolates after overnight culture in TSBGLU. Red arrows indicate cell sedimentation.

Supplementary Figure S2

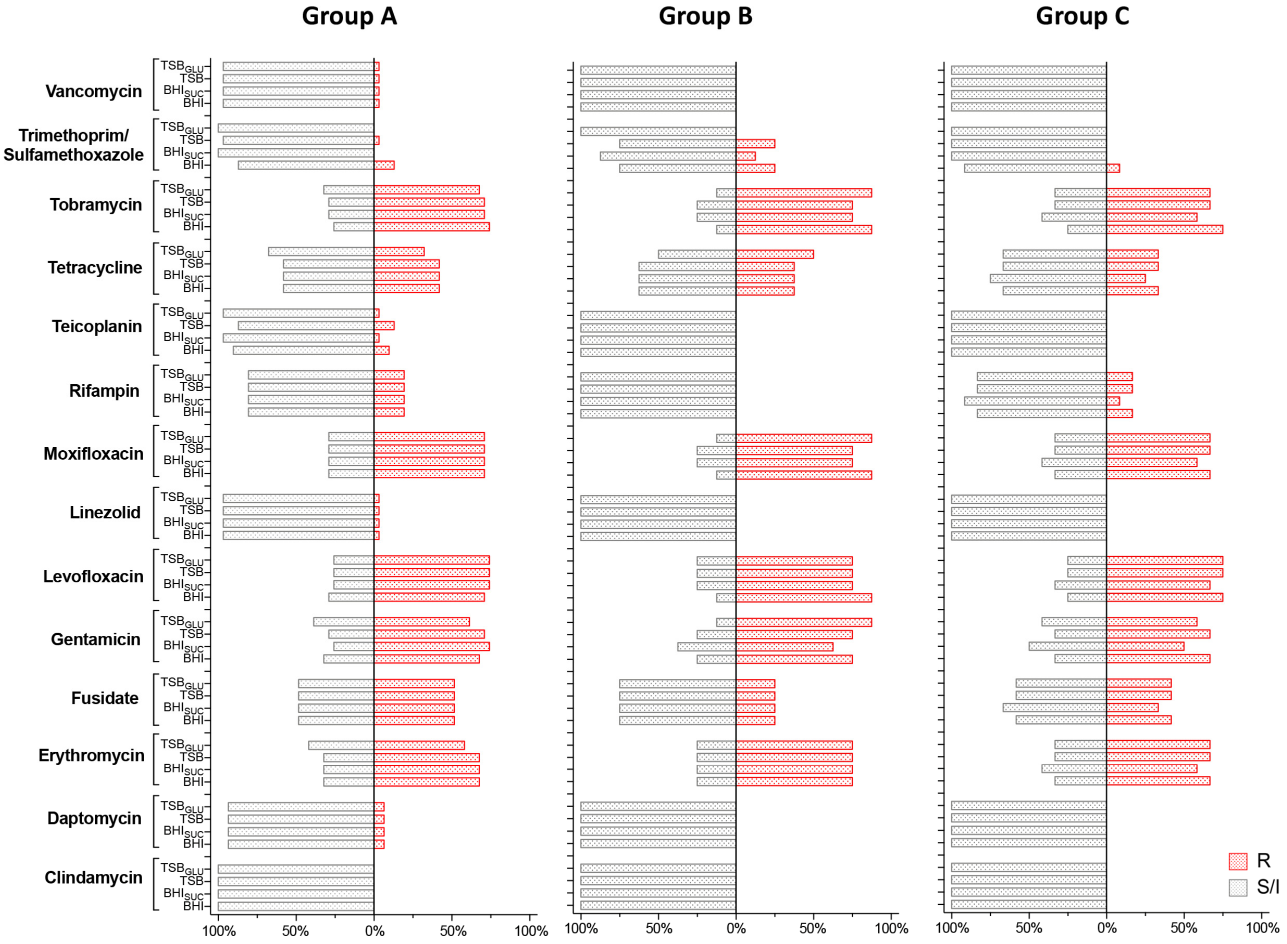

Figure S2: Relationships between antibiotic susceptibility and isolation source among *S. epidermidis* clinical strains.

Prevalence of antibiotic resistance among clinical *S. epidermidis* strains isolated from peripheral blood (group A), catheters (group B) or wounds (group C) grown in different indicated media. Statistical significance analized with Fischer exact test for each condition was reported (*p<0.05, **p<0.01, ***p<0.001). R= Resistant (in red), S/I= Susceptible or increased exposure (in grey).

Supplementary Figure S3

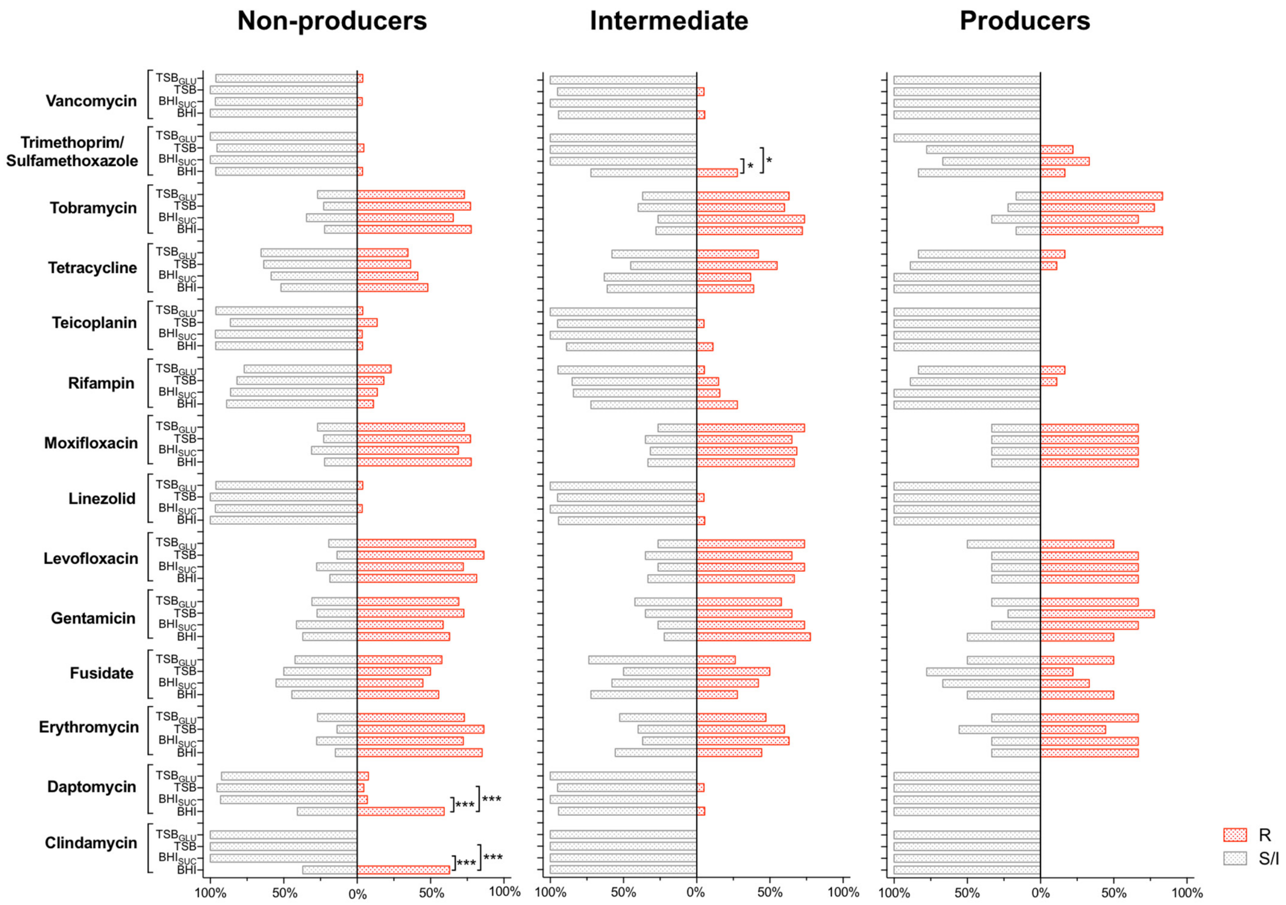

Figure S3: Relationships between antibiotic susceptibility and biofilm formation among *S. epidermidis* clinical strains.

Prevalence of antibiotic resistance among biofilm non-producer (Adhesion Index < 0.2), intermediate-producer (0.2 < Adhesion Index < 1) or biofilm-producer (Adhesion Index > 1) clinical *S. epidermidis* strains, grown in different indicated media. Statistical significance analized with Fischer exact test for each condition was reported (*p<0.05, **p<0.01, ***p<0.001). R= Resistant (in red), S/I= Susceptible (in grey) or increased exposure (in grey).

Supplementary Table S2

(A)

	Pearson's R	p value	AIC
M2	0.59	9.97E-22	355.417
M3	0.46	7.55E-13	392.211
M1	0.29	1.62E-05	401.397

(B)

	Estimate	SE	t value	Pr(>|t|)
(Intercept)	-0.514	0.175	-2.929	0.00379
Levofloxacin	-0.944	0.307	-3.075	0.00239
Linezolid	1.146	0.501	2.286	0.02330
Moxifloxacin	0.675	0.300	2.245	0.02585
Trimethoprim	0.591	0.198	2.981	0.00322

(A) Model comparison: condensed statistics about the three models ordered by increasing AIC (Akaike's information criterion). The Pearson's correlation refers to the comparison of predicted and observed values.
(B) Model M3: Antibiotics with a coefficient significantly different from 0. Direction of correlations correspond to the sign of the estimated parameters (first column).

Supplementary Information

Generalized Linear Models: All the three models significantly fit to the experimental data, but in different degrees as it can be appreciated from the correlation coefficients of the data points predicted by the model and the true ones (Table S1A). M2 is however a parameter-rich model, and its better fit compared to M1 may be a consequence of the extra number of free parameters. By exploiting the AIC of the three models, we can see that M2 has the minimum AIC value and therefore fits the data better. AIC values of different models can be compared to check how much the best model fits the data better than the other models. In this case the Akaike weight calculated as w=exp(–0.5 *(AIC_{M2}-AIC_{M1}) is 1.04e–10, implying that M2 is a much better model than M1; this means that the introduction of MIC values improves the model beyond the increased number of parameters. Analysis of association between biofilm and growth media, *i.e.*, the M1 model, highlights the existence of a significant negative association between biofilm formation and peripheral blood as isolation source (p=0.0021). In contrast, no significant association between growth medium and biofilm formation was detected for the other groups of isolates (catheters and wound). Since antibiotic resistance data are usually discretized on the basis of standardized EUCAST guidelines (e.g. Sensitive and Resistant), we also build the model M3, structurally similar to M2 but where the MICs are discretized. Therefore, M3 achieved intermediate performances, confirming that even partial knowledge of the antibiotic resistance patterns can be informative about biofilm formation.
